# Food brand recall and its association with diet-related behaviours among Thai children

**DOI:** 10.1017/S1368980026102183

**Published:** 2026-02-27

**Authors:** Nongnuch Jindarattanaporn, Salakjit Chuenchom

**Affiliations:** Institute for Population and Social Research, Mahidol University, Thailand

**Keywords:** Brand recall, Food, Purchasing, Consumption, Children

## Abstract

**Objective:**

Food brand marketing is one of the techniques used by the food industry to create positive images and enhance brand recall among children. The objectives of this study were to assess food and beverage brand recall and to examine the sociodemographic characteristics associated with food brand recall, as well as the purchasing and consumption of branded foods among Thai children.

**Design:**

Cross-sectional analysis of secondary data from the 2024 Monitoring on Food and Beverage Marketing to Children in Thailand survey. A validated and reliable questionnaire was used for data collection. Descriptive statistics were used to summarise sociodemographic characteristics, food and beverage brand recall, and purchasing behaviours. Associations between brand recall and purchasing behaviours were examined using multivariable logistic regression models.

**Setting:**

Eleven provinces across Thailand.

**Participants:**

A total of 2113 children aged 10–18 years.

**Results:**

Nearly 40 % of children recalled sweetened beverage brands, while 35 % recalled snack brands. Food and beverage brand recall was statistically associated with purchasing and consumption of branded foods across several product categories after adjustment for sociodemographic characteristics.

**Conclusions:**

Food and beverage brand recall was commonly reported among Thai children and showed statistically significant associations with purchasing and consumption behaviours. These findings contribute to the evidence base on food marketing exposure among children in Thailand.

In 2022, more than 390 million children and adolescents aged 5–19 years worldwide were classified as having overweight, including 160 million living with obesity^(1)^. According to the World Obesity Federation, the global prevalence of childhood obesity is projected to more than double by 2035. Among boys, the number of those with obesity is expected to rise from 140 million in 2020 to 208 million in 2035, while among girls, the number is projected to increase from 101 million to 175 million over the same period. Notably, the prevalence of obesity has been increasing more rapidly among children than adults^(2)^. In Thailand, childhood overweight and obesity remain a public health concern^(3)^. In 2023, 13·4 % of Thai children aged 6–14 years and 13·2 % of those aged 15–18 years were reported to be overweight or living with obesity^(4)^.

Unhealthy dietary patterns are commonly reported among children and are considered an important contributor to excess weight^(5)^. In Thailand, national data indicate increasing consumption of energy-dense foods among children^(6,7)^. Among children aged 6–14 years, the daily consumption of high-fat bakery products rose from 90·4 % in 2017 to 94·6 % in 2021. Over the same period, the proportion of children consuming fast food daily rose from 46·8 % to 64·8 %^(6,7)^. A national survey conducted in 2024 further reported that approximately nine out of 10 Thai children aged 6–18 years regularly consumed snacks and sugar-sweetened beverages, while eight out of 10 also consumed instant or ready-to-eat foods^(8)^. Beyond overall dietary patterns, increasing attention has been directed toward the role of food marketing environments in shaping children’s food-related exposure and experiences^(9)^.

Food brand marketing is widely used by the food industry to create positive brand images and enhance children’s ability to recognise and recall specific food brands^(10,11)^. Food brand recall, defined as the ability to remember branded food and beverage products, has been described as an important marker of children’s exposure to food marketing^(12)^. Previous studies from various countries have reported associations between food brand recall and children’s food-related preferences, purchasing behaviours and consumption patterns^(13,14)^. In addition, dietary behaviours established during childhood are often maintained later in life, highlighting the potential relevance of early food marketing exposure^(15)^.

In Thailand, empirical evidence on food brand recall among children remains limited. A previous study focusing on students aged 11–14 years reported an association between recognition food brands advertised in the media and the consumption of salty snacks and fast food^(16)^. However, this study was conducted more than a decade ago and was limited to Bangkok and Phetchaburi provinces, restricting its generalisability to the national child population^(16)^. Although Thailand has proposed draft legislation to regulate food marketing to children, the use of food branding is not explicitly addressed within this legislation^(17)^. Updated nationally representative evidence is therefore needed to better describe patterns of food brand recall and their associations with children’s food-related behaviours. The objectives of this study were to assess food and beverage brand recall among Thai children aged 10–18 years and to examine sociodemographic factors associated with brand recall, as well as the purchasing and consumption of branded foods.

## Methods

This cross-sectional study used secondary data from the Monitoring on food and beverage marketing to children in Thailand^(8)^. The survey was conducted nationwide across 11 provinces, including Bangkok, representing all regions of Thailand. Data collection was carried out between November 6, 2023, and February 15, 2024.

The study protocol was reviewed and approved by the Institutional Review Board of Institute for Population and Social Research at Mahidol University, Thailand on August 11, 2023. This observational study was not registered in a public registry. The study was conducted prior to the widespread adoption of study registration for observational research. However, the study protocol and analysis plan were developed a priori before data analysis.

### Reporting guideline

This study was reported in accordance with the Strengthening the Reporting of Observational Studies in Epidemiology (STROBE) guidelines. In addition, the STROBE extension for nutritional epidemiology (STROBE-nut) and the STROBE extension for abstracts were followed where applicable. The completed STROBE, STROBE-nut and STROBE-abstracts checklists are provided in the online supplementary material, Supplemental File 1.

### Participants

A nationally representative survey using multi-stage sampling design was conducted among children aged 6–18 years from eleven provinces across Thailand. The detailed sampling design and procedures have been described elsewhere^(8,18)^. A total of 4117 participants agreed to be interviewed. Monitoring on food and beverage marketing to children in Thailand did not assess brand recall among children aged 6-9 years since 2023–2024. At this early concrete operational stage, symbolic brand understanding and abstract emotional evaluation are limited, which may hinder accurate differentiation of brand exposure across contexts.^(19).^ Children in this age group were unable to comprehend the questions^(8)^. Therefore, the present analysis was restricted to children aged 10-18 years with complete brand recall data, resulting in a final analytic sample of 2113 participants.

### Measures

The questionnaire was developed in the Thai language, drawing on previous international surveys that examined the impacts of food marketing on children’s diet-related outcomes, and its development has been described in detail elsewhere^(20)^. The questionnaire included measures of the following variables:

### Food brand recall

Food brand recall was assessed using an open-ended question: ‘*What brand of food or drinks have you seen most often in the past week? This might include brands that you have seen in advertisements, shops or at your home*’. Participants reported the name of a food or beverage brand, which was subsequently categorised by the research team into seven groups: (1) fast food; (2) semi-processed foods (e.g. instant noodles, instant porridge, instant porridge); (3) confectionery (e.g. candies, gum, chocolate, jelly, custard, ice cream); (4) snacks (e.g. chips, crisp rice, corn chips, wafers, seaweed, salted nuts); (5) baked goods (e.g. raisin bread, chocolate cake, cookies, donuts); (6) sweetened beverages (e.g. carbonated drinks, fruit juices, chocolate malt beverages, sweetened soy milk); and (7) milk and dairy products (e.g. milk and yogurt).

### Diet-related behaviours

Diet-related behaviours referred to purchasing and consumption behaviours associated with the recalled food brand. Purchasing behaviour was assessed using a binary question asking whether the child ever used their own money to purchase food or drinks from the recalled brand (yes/no). Consumption frequency was assessed using an ordinal question: ‘*How often do you usually consume food or drinks from the brand you mentioned?*’ with three response categories: 1–3 d per week, 4–6 d per week, and every day (7 d).

### Sociodemographic characteristics

Sociodemographic variables included sex (male/female), age, place of residence (Bangkok, Central, North, South, Northeast), place of residence (urban/rural), educational level (illiterate, primary school, secondary school, or vocational certification) and daily pocket money for purchasing snacks and beverages.

Age was categorised into two groups (10–12 years and 13–18 years) based on the distribution of the sample, with approximately 60 % aged 10–12 years and 40 % aged 13–18 years.

Daily pocket money for purchasing snacks and beverages was categorised as ≤ 20 Thai baht (approximately ≤ USD 1·7 purchasing power parity), 21–50 Thai baht (approximately USD 1·8–4·3 purchasing power parity), > 50 Thai baht (approximately > USD 4·3 purchasing power parity).

### BMI

BMI was calculated as a function of weight and height. Weight was measured using a digital scale placed on a firm surface. Participants were instructed to remove their shoes and stand with both feet positioned at the centre of the scale, and their weight was recorded. Height was measured after shoes were removed, with participants standing upright against a wall with feet together. A tape measure was used to determine height, which was subsequently recorded.

The criteria of the International Obesity Task Force were applied to classify and describe overweight and obesity within the sample^(21,22)^. BMI was categorised as underweight (< 18·5), normal weight (18·5–22·9), and having overweight or obesity (23·0)^(21,22)^.

### Data collection procedure

Prior to data collection, twelve interviewers received standardised training on November 1, 2023, to ensure familiarity with the study protocol, questionnaire and ethical considerations. The session was facilitated by the principal investigator (NJ) and included practice interviews with children.

Data were obtained through face-to-face interviews with tablet computers running the Qualtrics offline survey application. Prior to fieldwork, households were contacted through village headpersons at the enumeration area level. Subsequently, data collection teams visited the sites, parental consent was sought from parents in all sampled households, and interviews were conducted with the children. Written informed consent was obtained from parents or guardians, and assent was obtained from children prior to enrolment in the study.

### Bias

To minimise information bias, a validated questionnaire was used, and interviewers received standardised training prior to fieldwork. The use of face-to-face interviews helped ensure comprehension of survey questions among children.

### Study size

The study size was determined by the original nationally representative survey designs. The present analysis included all eligible participants aged 10–18 years with complete data on food brand recall.

### Data analysis

All analyses were performed using SPSS version 18. Descriptive statistics were computed to summarise participants’ sociodemographic characteristics, food brand recall and food brand purchasing and consumption behaviours. Chi-square tests were performed to explore associations between variables (online supplementary material, Supplemental File 2) prior to conducting binary and ordinal logistic regression analyses.

Binary logistic regression models were used to examine associations between food brand recall and purchasing behaviour, while ordinal logistic regression models were applied to assess associations with consumption frequency. Multivariable models adjusted for potential confounders, including sex, age group, region, place of residence, educational level, daily pocket money and BMI. For ordinal logistic regression, the proportional odds assumption was assessed using the test of parallel lines.

There were no missing data for the variables included in the analyses; therefore, all eligible participants were included in the regression models. As a sensitivity analysis, age was additionally modelled as a continuous (ratio) variable instead of a categorical variable. The direction and magnitude of the associations remained consistent, and no substantive differences in the results were observed. A two-sided *P*-value of < 0·05 was considered statistically significant.

## Results

### Sociodemographic characteristics

Table 1 presents the sociodemographic characteristics of the 2113 participants included in the analysis. The sample consisted of 52 % females and 48 % males. The mean age of participants was 12·5 years (range: 10–18 years). Approximately half of the participants resided in urban areas. The majority of participants were classified as having a thin body weight status. Nearly three-fifths had attained primary school education. Almost half reported having a daily pocket money allowance of 21–50 Thai baht (approximately USD 1·8–4·3 purchasing power parity) for purchasing snacks and beverages. There were no missing data for variables included in the analysis.

**Table 1.** Sociodemographic characteristics of Thai children aged 10–18 years

Sociodemographic characteristics	All (*n* 2113)	
	Frequency	Percent
Gender		
Male	1011	47·8
Female	1102	52·2
Age (years): median 12; mean 12·5; (sd 2·2); range: 10–18		
10–12	1289	61·0
13–18	824	39·0
Regional area of residence		
Northeast	641	30·3
Central	556	26·3
South	361	17·1
North	319	15·1
Bangkok	236	11·2
Place of residence		
Urban	1064	50·4
Rural	1049	49·6
Educational level		
Illiterate	20	0·9
Primary school (grade 4–6)	1260	59·6
Secondary school (grade 7–12) or vocational certification	833	39·4
BMI: median 18·40; mean 19·72 (sd 4·92); range: 10·4–43·7		
Thinness (≤ 18·5)	1076	50·9
Normal (18·6–22·9)	583	27·6
Overweight or obese (≥ 23)	454	21·5
Pocket money per day for buying snacks and beverages: median 40; mean 45·38 (sd 29·20); range: 5–200-		
≤ 20 THB (approximately ≤ USD 1·7 PPP)	566	26·8
21–50 THB (approximately USD 1·8–4·3 PPP)	1011	47·8
> 50 THB (approximately > USD 4·3 PPP)	536	25·4

THB, Thai baht; PPP, purchasing power parity.

### Food brand recall

Table 2 summarises food and beverage brand recall among participants aged 10–18 years. Sweetened beverage brands were the most frequently recalled, reported by more than one-third of participants, followed by snack brands and semi-processed food brands. Detailed information on the specific brand names recalled is provided in the online supplementary material, Supplemental File 2.

**Table 2.** Prevalence of food and beverage brand recall among Thai children aged 10–18 years

Food brand recall	All (*n* 2113)	
	Frequency	Percent
1. Sweetened beverages	830	39·3
2. Snacks	740	35·0
3. Semi-processed foods	147	7·0
4. Fast food	142	6·7
5. Bakeries	118	5·6
6. Milk and dairy products	72	3·4
7. Confectionery	64	3·0

### Diet-related behaviours

Table 3 presents reported purchasing and consumption behaviours related to recalled food and beverage brands. Overall, 89·8 % of participants reported purchasing food or beverages from the recalled brand, while 10·2 % reported no purchasing. Regarding consumption frequency, 48·6 % reported consuming the recalled brands 1–3 d per week, followed by 35·3 % reported consumption on 4–6 d per week, and 16·1 % reported daily consumption.

**Table 3.** Diet-related behaviours among Thai children aged 10–18 years

Variables	All (*n* 2113)	
	Frequency	Percent
Purchasing behaviour		
Yes	1897	89·8
No	216	10·2
Eating behaviour		
1–3 d	1026	48·6
4–6 d	746	35·3
Every day (7 d)	341	16·1

### Association between food brand recall and diet-related behaviours

Table 4 presents the results of the multivariable regression analyses examining associations between food brand recall and diet-related behaviours. Regional area of residence, daily pocket money for purchasing snacks and beverages, and categories of recalled food brands were statistically associated with purchasing and consumption behaviours.

**Table 4.** Associations between food brand recall and purchasing and consumption behaviours among Thai children aged 10–18 years

Factors	food brand purchasing (*n* 2113)					Food brand consumption (*n* 2113)				
	Estimate	se	AOR	95 % CI	*P*-value	Estimate	se	COR	95 % CI	*P*-value
Gender										
Female	0·234	0·150	1·264	0·942, 1·697	0·119	0·105	0·084	1·111	0·942, 1·310	0·213
Male	Ref	–	–	–	–	Ref	–	–	–	–
Age										
10–12	0·229	0·436	1·258	0·535, 2·958	0·599	0·105	0·229	1·111	0·710, 1·739	0·645
13–18	Ref	–	–	–	–	Ref	–	–	–	–
Regional area of residence										
Bangkok	0·175	0·431	1·191	0·512, 2·772	0·685	**−**0·067	0·172	0·935	0·667, 1·311	0·698
Central	**−1·199**	**0·279**	**0·302**	**0·175, 0·521**	**< 0·001**	**0·725**	**0·143**	**2·064**	**1·561, 2·730**	**< 0·001**
Northeast	**−**0·363	0·275	0·695	0·406, 1·192	0·187	**−**0·056	0·131	0·945	0·732, 1·221	0·666
South	**−1·343**	**0·293**	**0·261**	**0·147, 0·463**	**< 0·001**	**0·398**	**0·153**	**1·489**	**1·103, 2·010**	**0·009**
North	Ref	–	–	–	–	Ref	–	–	–	–
Place of residence										
Urban	0·157	0·159	1·169	0·856, 1·597	0·325	0·057	0·092	1·058	0·883, 1·268	0·538
Rural	Ref	–	–	–	–	Ref	–	–	–	–
Educational level										
Illiterate	1·860	0·861	1·205	0·884, 3·666	0·998	0·006	0·430	1·006	0·433, 2·334	0·989
Primary school (grade 4–6)	**−**0·584	0·442	0·558	0·235, 1·326	0·186	0·076	0·230	1·079	0·688, 1·692	0·741
Secondary school (grade 7–12) or vocational certification	Ref	–	–	–	–	Ref	–	–	–	–
Pocket money per day for buying snacks and beverages										
≤ 20 THB (approximately ≤ USD 1·7 PPP)	**−0·565**	**0·235**	**0·568**	**0·359, 0·901**	**0·016**	0·224	0·129	1·251	0·971, 1·611	0·084
21–50 THB (approximately USD 1·8–4·3 PPP)	**−**0·336	0·210	0·715	0·474, 1·078	0·109	**0·222**	**0·108**	**1·249**	**1·010, 1·544**	**0·040**
> 50 THB (approximately >USD 4·3 PPP)	Ref	–	–	–	–	Ref	–	–	–	–
Food brand recall										
1. Fast food	**1·907**	**0·402**	**6·736**	**3·062, 14·817**	**< 0·001**	0·115	0·290	1·122	0·635, 1·982	0·692
2. Semi-processed foods	**1·743**	**0·248**	**5·714**	**3·514, 9·290**	**< 0·001**	0·485	0·284	1·623	0·930, 2·833	0·088
3. Snacks	**1·354**	**0·428**	**3·874**	**1·676, 8·958**	**0·002**	**0·586**	**0·249**	**1·798**	**1·105, 2·926**	**0·018**
4. Confectionery	**1·599**	**0·369**	**4·950**	**2·399, 10·211**	**< 0·001**	0·469	0·337	1·598	0·825, 3·095	0·164
5. Bakeries	**1·951**	**0·248**	**7·039**	**4·333, 11·434**	**< 0·001**	**0·747**	**0·296**	**2·111**	**1·182, 3·770**	**0·012**
6. Sweetened beverages	**1·178**	**0·407**	**3·248**	**1·463, 7·211**	**0·004**	**0·806**	**0·247**	**2·239**	**1·381, 3·630**	**0·001**
7. Milk and dairy products	Ref	**–**	**–**	**–**	**–**	Ref	**–**	**–**	**–**	**–**
Goodness of fit (Chi-square)	**–**	**–**	**–**	**–**	0·066	**–**	**–**	**–**	**–**	0·202
Test of parallel lines	**–**	**–**	**–**	**–**	**–**	**–**	**–**	**–**	**–**	0·102
Cutpoint 1 (1–3 d)	**–**	**–**	**–**	**–**	**–**	**−**0·113	0·514	0·893	0·326, 2·446	0·826
Cutpoint 2 (4–6 d)	**–**	**–**	**–**	**–**	**–**	1·642	0·515	5·166	1·881, 14·186	0·001

Note *n* = samples of this study; OR and 95 % CI are presented. OR > 1 indicates higher odds of purchasing or consuming foods or beverages based on recalled brands. AOR = adjusted OR; COR = cumulative OR; THB, Thai baht; PPP, purchasing power parity.

Ref = reference group. *P*-values < 0·05 were considered statistically significant.

Food brand purchasing: Pseudo R-Square (Nagelkerke) = 0·115.

Food brand consumption: Pseudo R-Square (Nagelkerke) = 0·145.

Boldface highlights the variables that remained significant in the final model.

Compared with participants who recalled milk and dairy product brands, those who recalled brands of fast food, semi-processed foods, snacks, confectionery, baked goods and sweetened beverages showed higher odds of purchasing the corresponding food or beverage categories. For consumption frequency, recalling snack brands was associated with higher cumulative odds of snack consumption compared with recalling milk and dairy product brands (cumulative OR = 1·798, 95 % CI: 1·105, 2·926). Similarly, recalling baked goods and sweetened beverage brands was associated with higher cumulative odds of consuming baked goods and sweetened beverages, respectively, compared with recalling milk and dairy product brands.

As a sensitivity analysis, age was modelled as a continuous variable instead of a categorical variable, and the overall pattern and direction of associations remained consistent with the main analysis (online supplementary material, Supplemental File 2).

## Discussion

This study examined food brand recall and assessed associations between sociodemographic characteristics, brand recall and purchasing and consumption behaviours related to recalled food brands among Thai children. Sweetened beverage and snack brands were most frequently recalled. Regional area of residence, daily pocket money, recall of snack, sweetened beverage and bakery brands were associated with reported purchasing and consumption of the corresponding branded products, whereas other sociodemographic characteristics showed no clear association.

This study found that snack and sweetened beverage brands were the most frequently recalled among Thai children. Our result was consistent with previous studies in Jamaica and the UK; sweetened beverage brands were among the most frequently recalled by children^(23,24)^. This pattern may reflect the high volume of marketing expenditure directed towards these product categories in Thailand, which exceeded that of other food categories in 2024, amounting to US$445 million^(25)^. Greater exposure to branded content has been associated with higher brand familiarity and recall in children^(26)^. These findings align with interpretive frameworks from learning theory, suggesting that repeated exposure to branded messages paired with engaging content may be associated with stronger emotional salience and memory of food brands^(27)^. For example, when children see commercials for crunchy snacks or sweetened beverages that include favourite celebrities or cartoons along with memorable tunes, their brains associate the food brand with positive feelings. This process leads to enhanced brand recall and the development of lasting food preferences deeply ingrained in the child’s mind over time^(28)^.

No significant associations were observed between gender or age and purchasing or consumption behaviours related to recalled food brands. This may indicate that sustained exposure to food branding occurs broadly across demographic subgroups. However, given the cross-sectional design, the study cannot clarify how such associations may vary over time^(29,30)^. Purchasing and consumption behaviours may also be shaped by contextual influences such as family environments and peer interactions, which were not captured in the present analysis^(31–33)^.

Regional differences were observed, with children residing in the Central and Southern regions more likely to report purchasing and consuming recalled food brands than those in the Northern region. These differences may reflect variations in urbanisation, availability of modern retail outlets, and density of food marketing environments across regions^(34–36)^. The Central region includes major commercial areas and the Eastern Economic Corridor^(34)^, while the area is characterised by tourism and modern trade expansion, potentially increasing children’s exposure to branded food products^(34)^. Consequently, children in these regions may have more frequent exposure to food brands, whereas the Northern region of Thailand is predominantly rural with less urban development compared to the Central and Southern regions^(36)^.

Children reporting lower daily pocket money allowances showed comparable likelihoods of purchasing recalled food brands relative to those with higher allowances. This finding suggests that brand recall was associated with purchasing behaviour regardless of daily spending capacity. Similar patterns have been reported in studies from Germany and the UK and other low- and middle-income countries such as India, where brand familiarity appears to be associated with children’s food choices even in contexts of limited purchasing power^(37–40)^.

Children who recalled sweetened beverage, snack and bakery brands were more likely to report purchasing and consuming these products than those who recalled milk and dairy brands. This pattern is consistent with evidence from the UK and the US indicating that recall of less healthy food brands is more prevalent and more strongly associated with reported consumption than recall of healthier products^(12,38,41)^. This may be due to the fact that milk is often consumed routinely without the need for brand recall, as it is provided by caregivers or through school programmes. In Thailand, milk is commonly provided through caregivers or school-based programmes, such as the National School Milk Programme^(42)^, which may limit opportunities for brand-based decision-making and reduce the relevance of brand recall for milk consumption.

A key strength of this study is the use of a nationally representative sample of Thai children aged 10–18 years, including children with limited literacy. Nevertheless, several limitations should be acknowledged. The cross-sectional design precludes causal inference, and the directionality of associations cannot be determined. Age was categorised for the main analyses, which may have obscured more nuanced age-related patterns; however, sensitivity analyses treating age as a continuous variable yielded consistent results. In addition, the study did not account for parental influences and peer dynamics^(33)^, frequency of brand exposure or attitudes towards brands, which may confound observed associations^(43,44)^. Future longitudinal research incorporating these factors would provide a more comprehensive understanding of how brand recall relates to children’s food-related behaviours over time.

The findings are likely generalisable to Thai children within similar food marketing and retail environments and may be relevant to other middle-income settings with pervasive food branding. The observed associations underscore the relevance of considering food branding as part of broader food marketing regulation. Incorporating provisions addressing food branding into existing policy frameworks may help reduce children’s exposure to branding strategies that are associated with unhealthy dietary patterns.

## Supporting information

Jindarattanaporn and Chuenchom supplementary material 1 Jindarattanaporn and Chuenchom supplementary material

Jindarattanaporn and Chuenchom supplementary material 2 Jindarattanaporn and Chuenchom supplementary material

## Supplementary material

For supplementary material accompanying this paper, visit https://doi.org/10.1017/S1368980026102183
