# Supplementary material for: Food brand recall and its association with diet-related behaviours among Thai children
Source: Public Health Nutr. 2026 Feb 27;29(1):e63. doi: 10.1017/S1368980026102183 (PMC13087976; doi:10.1017/S1368980026102183)
Supplement: Jindarattanaporn and Chuenchom supplementary material 2 — Jindarattanaporn and Chuenchom supplementary material [file S1368980026102183sup002.docx]

**Supplementary 2**

The univariate analysis was conducted by using Chi-square to analyze the associations between socio-demographic characteristics, unhealthy food brand recall and behaviors of Thai children aged 10-18 years. Table A1-A2 shows the factors relate to purchasing behavior, and eating behavior.

Table A1. Factors associate with purchasing behavior among Thai children aged 10-18 years.

| **Variables** | **All (n=2,113)** | **Percentage of purchasing (n=2,113)** | | **P-value** |
| --- | --- | --- | --- | --- |
|  |  | **Yes** | **No** |  |
| **Gender** |  |  |  |  |
| Male | 1,011 | 88.8 | 11.2 | 0.165 |
| Female | 1,102 | 90.7 | 9.3 |  |
| **Age** |  |  |  |  |
| 10-12 | 1,289 | 87.8 | 12.2 | <0.001 |
| 13-18 | 824 | 92.8 | 7.2 |  |
| **Regional area of residence** |  |  |  |  |
| Bangkok | 236 | 96.2 | 3.8 | <0.001 |
| Central | 556 | 86.3 | 13.7 |  |
| Northeast | 641 | 91.6 | 8.4 |  |
| South | 361 | 84.8 | 15.2 |  |
| North | 319 | 93.1 | 6.9 |  |
| **Place of residence** |  |  |  |  |
| Urban | 1,064 | 91.0 | 9.0 | 0.067 |
| Rural | 1,049 | 88.6 | 11.4 |  |
| **Educational level** |  |  |  |  |
| Illiterate | 20 | 100.0 | 0.0 | <0.001 |
| Primary school (grade 4-6) | 1,260 | 87.5 | 12.5 |  |
| Secondary school (grade 7-12) or vocational certification | 833 | 93.0 | 7.0 |  |
| **BMI** |  |  |  |  |
| Thinness (≤18.5) | 1,076 | 90.1 | 9.9 | 0.228 |
| Normal (18.6-22.9) | 583 | 90.7 | 9.3 |  |
| Overweight/obese (≥23) | 454 | 87.7 | 12.3 |  |
| **Pocket money per day for buying snacks and beverages** |  |  |  |  |
| ≤ 20 baht | 566 | 87.1 | 12.9 | 0.020 |
| 21-50 baht | 1,011 | 90.0 | 10.0 |  |
| > 50 baht | 536 | 92.2 | 7.8 |  |
| **Food brand recall** |  |  |  |  |
| 1. Fast food | 142 | 72.5 | 27.5 | <0.001 |
| 1. Semi-processed foods | 147 | 93.9 | 6.1 |  |
| 1. Snacks | 740 | 89.9 | 10.1 |  |
| 1. Confectionaries | 64 | 85.9 | 14.1 |  |
| 1. Bakeries | 118 | 89.0 | 11.0 |  |
| 1. Sweetened beverages | 830 | 92.7 | 7.3 |  |
| 1. Milk and dairy products | 72 | 86.1 | 13.9 |  |

Table A2. Factors associate with eating behavior among Thai children aged 10-18 years.

| **Variables** | **All (n=2,113)** | **Percentage of frequency of eating (n=2,113)** | | | **P-value** |
| --- | --- | --- | --- | --- | --- |
|  |  | **1-3 days** | **4-6 days** | **Every day (7 days)** |  |
| **Gender** |  |  |  |  |  |
| Male | 1,011 | 47.2 | 35.5 | 17.3 | 0.297 |
| Female | 1,102 | 49.8 | 35.1 | 15.1 |  |
| **Age** |  |  |  |  |  |
| 10-12 | 1,289 | 50.1 | 33.0 | 16.8 | 0.025 |
| 13-18 | 824 | 46.1 | 38.8 | 15.0 |  |
| **Regional area of residence** |  |  |  |  |  |
| Bangkok | 236 | 38.1 | 47.0 | 14.8 | <0.001 |
| Central | 556 | 59.7 | 29.1 | 11.2 |  |
| Northeast | 641 | 43.2 | 35.9 | 20.9 |  |
| South | 361 | 51.8 | 33.8 | 14.4 |  |
| North | 319 | 43.9 | 37.9 | 18.2 |  |
| **Place of residence** |  |  |  |  |  |
| Urban | 1,064 | 48.9 | 35.8 | 15.3 | 0.581 |
| Rural | 1,049 | 48.2 | 34.8 | 17.0 |  |
| **Educational level** |  |  |  |  |  |
| Illiterate | 20 | 45.0 | 40.0 | 15.0 | 0.042 |
| Primary school (grade 4-6) | 1,260 | 50.5 | 32.6 | 16.9 |  |
| Secondary school (grade 7-12) or vocational certification | 833 | 45.7 | 39.3 | 15.0 |  |
| **BMI** |  |  |  |  |  |
| Thinness (≤18.5) | 1,076 | 48.6 | 35.1 | 16.3 | 0.158 |
| Normal (18.6-22.9) | 583 | 45.1 | 37.2 | 17.7 |  |
| Overweight/obese (≥23) | 454 | 52.9 | 33.3 | 13.9 |  |
| **Pocket money per day for buying snacks and beverages** |  |  |  |  |  |
| ≤ 20 baht | 566 | 49.8 | 32.0 | 18.2 | 0.190 |
| 21-50 baht | 1,011 | 49.2 | 35.6 | 15.2 |  |
| > 50 baht | 536 | 46.1 | 38.2 | 15.7 |  |
| **Food brand recall** |  |  |  |  |  |
| 1. Fast food | 142 | 57.7 | 29.6 | 12.7 | 0.015 |
| 1. Semi-processed foods | 147 | 48.3 | 39.5 | 12.2 |  |
| 1. Snacks | 740 | 51.1 | 33.2 | 15.7 |  |
| 1. Confectionaries | 64 | 51.6 | 35.9 | 12.5 |  |
| 1. Bakeries | 118 | 47.5 | 34.7 | 17.8 |  |
| 1. Sweetened beverages | 830 | 43.6 | 37.7 | 18.7 |  |
| 1. Milk and dairy products | 72 | 61.1 | 31.9 | 6.9 |  |

Table A3 presents data on food brand recall of participants aged 10-18 years. Overall, the most frequently recalled food brand was Lays (savory snack brand), followed by Coke (carbonated beverage brand) and Oishi (beverage brand).

Table A3. Food brand recall among Thai Children aged 10-18 Years

| **Variables** | **Number (%) of a food brand recall (n=2,113)** | | | | | | | |
| --- | --- | --- | --- | --- | --- | --- | --- | --- |
| **Brand** | **n** | **%** | **Brand** | **n** | **%** | **Brand** | **n** | **%** |
| Lays | 524 | 24.8 | Sprite | 9 | 0.4 | Bear Milk | 2 | 0.1 |
| Coke | 221 | 10.5 | Twistko | 9 | 0.4 | Big Bloom | 2 | 0.1 |
| Oishi | 204 | 9.7 | Big Cola | 8 | 0.4 | Birdy | 2 | 0.1 |
| Pepsi | 180 | 8.5 | Dinopark | 8 | 0.4 | Chocopie | 2 | 0.1 |
| Mama | 128 | 6.1 | Meiji | 8 | 0.4 | Fun-O | 2 | 0.1 |
| KFC | 120 | 5.7 | Bento | 7 | 0.3 | Karada | 2 | 0.1 |
| Bengbeng | 44 | 2.1 | Jaxx | 7 | 0.3 | Lotus | 2 | 0.1 |
| Walls | 44 | 2.1 | KitKat | 7 | 0.3 | Mashita | 2 | 0.1 |
| Snack Jack | 42 | 2.0 | Kohkae | 7 | 0.3 | Misterdonut | 2 | 0.1 |
| Taokaenoi | 40 | 1.9 | Foremost | 6 | 0.3 | Nescafe | 2 | 0.1 |
| Milo | 35 | 1.7 | Alfie | 5 | 0.2 | Nissin | 2 | 0.1 |
| Ovaltine | 34 | 1.6 | Sunbites | 5 | 0.2 | Paprika | 2 | 0.1 |
| Ichitan | 33 | 1.6 | Tawan | 5 | 0.2 | Bon O Bon | 1 | 0.0 |
| Lactasoy | 29 | 1.4 | YenYen | 5 | 0.2 | BunBun | 1 | 0.0 |
| Pipo | 21 | 1.0 | Doritos | 4 | 0.2 | Choki Choki | 1 | 0.0 |
| Fanta | 20 | 0.9 | Hanami | 4 | 0.2 | JollyBears | 1 | 0.0 |
| Yakult | 20 | 0.9 | Malee | 4 | 0.2 | Kellogg's | 1 | 0.0 |
| Campus | 19 | 0.9 | Pocky | 4 | 0.2 | Kinder joy | 1 | 0.0 |
| Betagen | 16 | 0.8 | Potae | 4 | 0.2 | Kuga | 1 | 0.0 |
| Dutch Mill | 16 | 0.8 | Cheetos | 3 | 0.1 | Lepang | 1 | 0.0 |
| Deedo | 15 | 0.7 | Cornae | 3 | 0.1 | Lipton | 1 | 0.0 |
| Est | 13 | 0.6 | D-malt | 3 | 0.1 | M&M | 1 | 0.0 |
| Hersheys | 13 | 0.6 | D-na | 3 | 0.1 | Melody | 1 | 0.0 |
| Taro | 13 | 0.6 | Gato | 3 | 0.1 | Pizza Hut | 1 | 0.0 |
| Testo | 13 | 0.6 | Heartbeat | 3 | 0.1 | PR Big Bag | 1 | 0.0 |
| Yumyum | 13 | 0.6 | Knorr | 3 | 0.1 | Pringles | 1 | 0.0 |
| Farmhouse | 11 | 0.5 | McDonald | 3 | 0.1 | Roller Coaster | 1 | 0.0 |
| PuThai | 10 | 0.5 | Ole | 3 | 0.1 | Sponsor | 1 | 0.0 |
| Ezygo | 9 | 0.4 | Sunsu | 3 | 0.1 | Twisty | 1 | 0.0 |
| Oreo | 9 | 0.4 | Thaidenmark | 3 | 0.1 | Unif | 1 | 0.0 |
| Pizza company | 9 | 0.4 | Waiwai | 3 | 0.1 | Vitamilk | 1 | 0.0 |

Table A4 presents sensitivity analyses in which age was modelled as a continuous variable to assess the robustness of the main findings. The direction and statistical significance of associations between food brand recall and both purchasing and consumption behaviours were consistent with the primary analyses, and the proportional odds assumption was satisfied.

Table A4. Sensitivity analysis examining associations between food brand recall and food brand purchasing and consumption, with age modelled as a continuous variable (n = 2,113)

| **Factors** | **food brand purchasing**  **(n=2,113)** | | | | | **Food brand consumption**  **(n=2,113)** | | | | |
| --- | --- | --- | --- | --- | --- | --- | --- | --- | --- | --- |
|  | **Estimate** | **s.e.** | **AOR** | **95% CI** | **P-value** | **Estimate** | **s.e.** | **COR** | **95% CI** | **P-value** |
| **Gender** |  |  |  |  |  |  |  |  |  |  |
| Female | -0.232 | 0.150 | 0.793 | 0.591-1.065 | 0.123 | 0.106 | 0.084 | 1.112 | 0.943-1.311 | 0.209 |
| Male | Ref | - | - | - | - | Ref | - | - | - | - |
| **Age** | 0.034 | 0.066 | 0.610 | 0.909-1.176 | 0.610 | .008 | .346 | 0.737 | 0.671-1.687 | 0.982 |
| **Regional area of residence** |  |  |  |  |  |  |  |  |  |  |
| Bangkok | 0.158 | 0.431 | 1.171 | 0.503-2.725 | 0.714 | 0.061 | 0.172 | 1.063 | 0.759-1.488 | 0.723 |
| Central | **-1.207** | **0.279** | **0.299** | **0.173-0.516** | **<0.001** | **-0.729** | **0.142** | **0.483** | **0.365-0.638** | **<0.001** |
| Northeast | -0.375 | 0.275 | 0.688 | 0.401-1.178 | 0.173 | 0.051 | 0.130 | 1.052 | 0.816-1.358 | 0.694 |
| South | **-1.353** | **0.293** | **0.258** | **0.146-0.458** | **<0.001** | **-0.403** | **0.153** | **0.668** | **0.495-0.901** | **0.008** |
| North | Ref | - | - | - | - | Ref | - | - | - | - |
| **Place of residence** |  |  |  |  |  |  |  |  |  |  |
| Urban | 0.160 | 0.159 | 1.174 | 0.860-1.603 | 0.314 | 0.057 | 0.092 | 0.945 | 0.789-1.132 | 0.539 |
| Rural | Ref | - | - | - | - | Ref | - | - | - | - |
| **Educational level** |  |  |  |  |  |  |  |  |  |  |
| Illiterate | 1.860 | 0.861 | 1.188 | 0.899-3.887 | 0.998 | -0.009 | 0.430 | 0.991 | 0.983 | 0.983 |
| Primary school (grade 4-6) | **-**0.256 | 0.288 | 0.774 | 0.440-1.362 | 0.374 | 0.021 | 0.093 | 1.021 | 0.823 | 0.823 |
| Secondary school (grade 7-12) or vocational certification | Ref | - | - | - | - | Ref | - | - | - | - |
| **Pocket money** **per day for buying snacks and beverages** |  |  |  |  |  |  |  |  |  |  |
| ≤ 20 baht | **-.0546** | **0.237** | **0.579** | **0.364-0.921** | **0.021** | 0.223 | 0.129 | 0.800 | 0.621-1.031 | 0.084 |
| 21–50 baht | -0.321 | 0.211 | 0.725 | 0.480-1.097 | .128 | **0.222** | **0.108** | **0.801** | **0.648-0.991** | **0.041** |
| > 50 baht | Ref | - | - | - | - | Ref | - | - | - | - |
| **Food brand recall** |  |  |  |  |  |  |  |  |  |  |
| 1.Fast food | **1.914** | **.402** | **6.783** | **3.084-14.918** | **<0.001** | 0.113 | 0.290 | 1.119 | 0.636-1.992 | 0.698 |
| 2.Semi-processed foods | **1.744** | **.248** | **5.723** | **3.521-9.302** | **<0.001** | 0.485 | 0.284 | 1.624 | 0.936-2.857 | 0.088 |
| 3.Snacks | **1.351** | **.428** | **3.861** | **1.669-8.928** | **0.002** | **0.586** | **0.249** | **1.797** | **1.114-2.960** | **0.018** |
| 4.Confectionery | **1.618** | **.370** | **5.041** | **2.443-10.401** | **<0.001** | 0.467 | 0.337 | 1.596 | 0.824-3.101 | 0.166 |
| 5.Bakeries | **1.955** | **.247** | **7.063** | **4.349-11.427** | **<0.001** | **0.750** | **0.296** | **2.118** | **1.192-3.811** | **0.011** |
| 6.Sweetened beverages | **1.178** | **.407** | **3.249** | **1.463-7.217** | **0.004** | **0.805** | **0.247** | **2.237** | **1.392-3.671** | **0.001** |
| 7.Milk and dairy products | Ref | **-** | **-** | **-** | **-** | Ref | **-** | **-** | **-** | **-** |
| Goodness of fit  (Chi-square) | **-** | **-** | **-** | **-** | 0.056 | **-** | **-** | **-** | **-** | 0.779 |
| Test of parallel lines | **-** | **-** | **-** | **-** | **-** | **-** | **-** | **-** | **-** | 0.130 |
| Cutpoint 1 (1–3 days) | **-** | **-** | **-** | **-** | **-** | 0.192 | 0.274 | 1.212 | 0.714-2.093 | 0. 483 |
| Cutpoint 2 (4–6 days) | **-** | **-** | **-** | **-** | **-** | 1.947 | 0.277 | 7.008 | 4.100-12.194 | <0.001 |

*Note* Food brand purchasing: Pseudo R-Square (Nagelkerke) = 0.115

Food brand consumption: Pseudo R-Square (Nagelkerke) = 0.152
